# Kidney Biopsy and Immuno-Rheumatological Diseases: A Retrospective and Observational Study

**DOI:** 10.3390/jpm14010092

**Published:** 2024-01-13

**Authors:** Antonietta Gigante, Rosario Cianci, Annalisa Villa, Chiara Pellicano, Konstantinos Giannakakis, Edoardo Rosato, Francesca Romana Spinelli, Umberto Basile, Cosimo Racco, Elena Maria Di Virgilio, Bruna Cerbelli, Fabrizio Conti

**Affiliations:** 1 Department of Translational and Precision Medicine, Sapienza University of Rome, 00185 Rome, Italy; antonietta.gigante@uniroma1.it (A.G.); rosario.cianci@uniroma1.it (R.C.); annalisa.villa@uniroma1.it (A.V.); chiara.pellicano@uniroma1.it (C.P.); edoardo.rosato@uniroma1.it (E.R.); divirgilio.1742532@studenti.uniroma1.it (E.M.D.V.); 2 Department of Radiological, Oncological and Pathological Sciences, Sapienza University of Rome, 00185 Rome, Italy; konstantinos.giannakakis@uniroma1.it; 3 Rheumatology Unit, Department of Clinical Internal, Anaesthesiological and Cardiovascular Sciences, Sapienza University of Rome, 00185 Rome, Italyfabrizio.conti@uniroma1.it (F.C.); 4 Department of Clinical Pathology, Santa Maria Goretti Hospital, AUSL Latina, 04100 Latina, Italy; c.racco@ausl.latina.it; 5 Department of Medico-Surgical Sciences and Biotechnologies, Sapienza University of Rome, 04100 Latina, Italy; bruna.cerbelli@uniroma1.it

**Keywords:** kidney biopsy, autoimmunity, immuno-rheumatological diseases, glomerulonephritis, vasculitis, lupus nephritis

## Abstract

Renal involvement is a common occurrence in patients with immuno-rheumatological diseases (IRDs). Several instances of glomerulonephritis (GN) occur in the setting of IRD and complicate the clinical course of an underlying condition. The aim of this study was to observe the spectrum of nephropathies according to age, kidney function, history of IRD at the time of biopsy, and histopathological kidney diagnosis. We evaluated data relating to 699 consecutive kidney native biopsies (female 52.1%) with a median age of 48 years (IQR 34–62) performed in adult patients collected over 15 years. The study population was divided into three groups: patients with kidney histological findings correlated to underlying IRD (Group 1), patients with kidney histological findings not correlated to underlying IRD (Group 2), and patients with kidney histological findings compatible with “de novo” IRD (absent in personal medical history) (Group 3). Kidney involvement related to IRD was found in 25.2% of patients. Group 1 was mostly represented by lupus nephritis (76.6%), with a younger age than Group 3 (*p* < 0.001) and by a higher percentage of females than other groups (*p* < 0.001). Group 3 was the most represented by microscopic polyangiitis (50.8%) when compared with the other two groups (*p* < 0.001). Acute nephritic syndrome (*p* < 0.001), acute kidney injury (AKI), and abnormal urinalysis (*p* < 0.001) were more represented in Group 3 than the other groups. In conclusion, IRDs are characterized by different clinical presentations and heterogeneous histological findings. Kidney biopsy remains fundamental to achieving the correct diagnosis and starting targeted therapy.

## 1. Introduction

All kidney structures can be affected by immuno-rheumatological diseases (IRDs). Glomeruli, vessels, tubuli and interstitium can be both targets of autoimmune processes and damaged [1].

Kidney injury linked to IRD is related to a disturbance of immunity with loss of tolerance to normal cells.

Different mechanisms may be involved in the determinism of kidney immunological damage, such as antibodies’ mediated mechanisms against autoantigens outside the kidney, the complement cascade with a central role in the immune response, the deposition of immune complexes that originate within the kidney, and antigens and antibodies being neither derived nor deposited within the kidneys [2].

Diabetes and hypertension are the most common and known causes of kidney diseases [3] while other causes are represented by glomerulonephritis (GN), often with depositions of immune complexes in different renal compartments. However, it must be clarified that not all forms of GN are attributable to immune mechanisms. In addition to GN, several forms of tubulointerstitial diseases are correlated to immune mechanisms with significantly lower prevalence [1].

The medical history and clinical presentation are not sufficient to establish the diagnosis and, thus, an invasive procedure, with a 5% risk of major post-biopsy complications, is mandatory [4].

Kidney biopsy plays a pivotal role in providing an accurate diagnosis of kidney disease, especially GN, which is necessary for starting targeted therapy.

In clinical practice, a kidney biopsy is not performed if the diagnosis can be made without histology, for example, in the course of long-standing diabetes with slow progression towards proteinuria or chronic kidney diseases (CKDs) or acute tubular necrosis.

Glomerulonephritis, classified as primary and secondary, accounts for 25% of the cases of kidney failure requiring kidney replacement therapy (KRT) [5]. Secondary GN affects patients of all ages and is mainly characterized by immune-mediated damage to the basement membrane, the mesangium, or the capillary endothelium [6]. Usually, secondary GN occurs in the setting of autoimmune diseases, infections (HIV, hepatitis, etc.), or malignancy (which includes both solid and hematological malignancy).

The hallmarks of the clinical presentation of GN are proteinuria, hematuria, acute kidney injury (AKI), or CKD.

Based on specific variables, such as the onset of clinical presentation and the diseases associated, GN can be manifested with nephrotic syndrome (NS), acute nephritic syndrome (ANS), rapidly progressive renal injury, and urinary abnormalities (UAs) [7].

Kidney involvement represents one of the major issues in many IRDs, with variable occurrence in Sjögren syndrome (SS), polymyositis, dermatomyositis, systemic sclerosis (SSc), rheumatoid arthritis (RA), and antiphospholipid syndrome (APS) and with a prevalence of approximately 50% in cases of systemic lupus erythematosus (SLE) [8].

The mechanism for kidney injury is based on antibodies’ deposition on renal cells and their exposed fragment crystalline regions that can recruit inflammatory cells (T-cells, neutrophils, macrophages, platelets, and soluble mediators) with likely complement activation [1]. The response of the cell residence plays an important role in determining the type of inflammation and clinical signs and symptoms.

If the damage is not promptly discovered and treated, the evolution towards fibrosis can lead to end-stage kidney disease.

Changes in kidney function and specific histological findings, such as crescent formation, can be of significant prognostic value that often leads to a specific systemic therapy.

With this background the aim of this study was to observe the spectrum of nephropathies according to age, kidney function, history of IRD at the time of biopsy, and histopathological kidney diagnosis.

## 2. Materials and Methods

We evaluated data relating to 728 consecutive renal native biopsies performed in adult patients at Policlinico Umberto I of Rome (Italy), collected during the years ranging from 2000 to 2015. Data, about 699, were included in the present study: data from 29 renal biopsies were not included in the present analysis (non-diagnostic for inadequate sampling of materials, normal renal tissue, kidney tumors, or transplantation).

All data were provided by the wards where the patients were admitted with an indication to perform a kidney biopsy.

Kidney biopsies were performed for common indications, including NS, ANS, AKI, and UA.

We considered age, gender, and clinical presentation defined as UA (i.e., hematuria and/or proteinuria not in the nephrotic range), NS, ANS, AKI, or rapidly progressive renal injury.

From the clinical records, we collected clinical information including comorbidities and biochemical analyses. We recorded the history of IRD, cardiovascular disease, systemic arterial hypertension, hematological diseases, non-steroidal anti-inflammatory drugs (NSAIDs), and other nephrotoxic drugs.

The presence and stage of CKD were characterized according to Kidney Disease Outcomes Quality Initiative (K-DOQI) guidelines if the kidney damage or estimated glomerular filtration rate (eGFR) was <60 mL/min, persisting for 3 months or more in association with ultrasound abnormalities [9]. Kidney function was defined by eGFR considering creatinine values at admission in the hospital. To estimate eGFR, the Chronic Kidney Disease Epidemiology Collaboration (CKD-EPI) equation was used as follows: eGFR = 142 × min(Scr/κ, 1)α × max(Scr/κ, 1) − 1.200 × 0.9938 Age × 1.012 [if female]; where Scr is standardized serum creatinine in mg/dL, κ is 0.7 for females or 0.9 for males, α is −0.241 for females or −0.302 for males, min(Scr/κ, 1) is the minimum of Scr/κ or 1.0, and max(Scr/κ, 1) is the maximum of Scr/κ or 1.0 [10].

Acute kidney injury was defined according to Kidney Disease Improving Global Outcomes (KDIGO) guidelines as any of the following: increase in serum creatinine by ≥0.3 mg/dL within 48 h; increase in serum creatinine to ≥1.5 times baseline, which is known or presumed to have occurred within the prior 7 days; or urine volume < 0.5 mL/kg/h for 6 h [11].

Nephrotic syndrome and ANS were diagnosed according to KDIGO practice guidelines based on the Glomerular Diseases Work Group as follows:

- NS: peripheral oedema, hypoalbuminemia, hyperlipidemia, and proteinuria > 3.5 g/24 h;
- ANS: haematuria, mild proteinuria (<3.5 g/24 h), arterial hypertension, and reduced GFR [7].

Kidney diseases were divided into four major categories: (1) primary GN; (2) secondary GN; (3) tubulointerstitial nephropathies (TIN); and (4) vascular nephropathies (VN).

IgA nephropathy (IgAN), membranous GN (MGN), focal segmental glomerulosclerosis (FSGS), minimal change disease (MCD), and post-streptococcal GN were considered primary GN.

Secondary GN was considered as follows:

- Immune-mediated GN as SLE; Schonlein–Henoch purpura; vasculitis, such as cryoglobulinemia vasculitis (CV); microscopic polyangiitis (MPA); granulomatosis with polyangiitis (GPA); eosinophilic granulomatosis with polyangiitis (Churg–Strauss); polyarteritis nodosa (PAN); and APS;
- Metabolic and hereditary-disorder-associated GN, such as diabetes mellitus, Alport’s syndrome, and Anderson–Fabry disease;
- Hematological disease-associated renal involvement, such as monoclonal gammopathy, Waldenstrom’s macroglobulinemia, light-chain disease, and amyloidosis;
- Chronic TIN (CTIN), acute TIN (ATIN), multiple myeloma-associated TIN, and IgG4-positive TIN.

Vascular nephropathies were considered as follows: thrombotic microangiopathy (TMA), nephroangiosclerosis (NAS), and scleroderma renal crisis (SRC).

In particular, TMA is characterized by microvascular thrombosis formation with tissue ischemia; NAS is characterized by thickening and intimal fibrosis of medium- and small-sized arteries, arteriolar thickening, hyalinosis, and glomerular sclerosis with tubulointerstitial fibrosis; and SRC is characterized by myxoid intimal proliferation with luminal narrowing, collagen deposition in the intima, and smooth muscle cell infiltration resulting in the “onion skin” finding.

## 3. Renal Biopsy and Histology

Renal tissue was obtained by a percutaneous needle biopsy with ultrasound guidance (Aplio Ultrasound System SSA-790 with convex 3.5 MHz probe, Toshiba, Tokyo, Japan). Tissue cores were received within 15 min of biopsy and divided into three portions for immunofluorescence, light, and electron microscopy. Multiple paraffin sections were stained with periodic acid–Schiff (PAS), hematoxylin and eosin, and PAS–silver methenamine. All biopsy samples had at least 10 glomeruli. Immunofluorescence was performed on 5 mm cryostat sections using polyclonal fluorescein isothiocyanate-conjugated antibodies, such as IgG, IgM, IgA, C3, C1q, kappa, lambda, and fibrinogen (Dako, Carpinteria, CA, USA). The severity of glomerular lesions was graded semi-quantitatively on a scale of 0 to 3 according to the percentage of glomerular involvement: 0, absence of lesions; 1, involvement of 1–30% of specimen/total number of glomeruli; 2, involvement of 31–60% of specimen/total number of glomeruli; and 3, involvement of 61–100% of specimen/total number of glomeruli [12]. The following glomerular lesions were evaluated: karyorrhexis/fibrinoid necrosis, leukocyte exudation, cellular and fibrous crescents, and segmental/global sclerosis. The tubular lesions evaluated were tubulitis and tubular atrophy; the interstitial lesions evaluated were inflammatory infiltration and fibrosis. For arteriolosclerosis, we examined the percentage of vessels showing hyaline change or wall thickening. Wall thickening was evaluated as the ratio of the luminal diameter to the outer diameter and defined as a ratio less than 0.5 [13]. A pathologist without prior knowledge of any information concerning each patient performed these morphological evaluations.

This study was conducted in accordance with the Declaration of Helsinki. All the patients have received and signed the informed consent forms. This study project was approved by the Local Ethics Committee.

## 4. Statistical Analysis

SPSS version 26.0 software (Bioz, Los Altos, CA, USA) was used for statistical analysis. The Shapiro–Wilk test was used to evaluate the normal distribution of data. Categorical variables were expressed as absolute frequencies and percentages of the total. Continuous variables were expressed as the median and interquartile range (IQR). Differences between groups were evaluated by the Student’s or Mann–Whitney’s *t*-test. Differences between categorical variables were evaluated by the chi-square or Fisher’s exact test. The Pearson or Spearman correlation test was used for bivariate correlations. A *p*-value < 0.05 was considered significant.

## 5. Results

Demographic characteristics, risk factors for kidney damage, and urinary signs were registered. In the study population, 364 (52.1%) were females and the median age was 48 years (IQR 34–62). The median value of serum creatinine was 1.20 mg/dL (IQR 0.80–2.30), the median blood urea level was 41 mg/dL (IQR 23–70), the median eGFR was 54 mL/min/1.73 m^2^ (IQR 26–88), and the median proteinuria value was 2.00 g/24 h (IQR 0.85–4.80).

Urinary abnormalities were found in 280 patients (40.1% of the population) while 249 patients (35.6%) presented isolated hematuria. Thus, ANS was found in 92 patients (13.2%) and NS in 235 patients (33.6%).

Acute kidney injury was registered in 147 patients (21% of the cohort), CKD in 172 patients (24.6%), and AKI in a pre-existent CKD condition was found in 54 patients (7.7%). According to comorbidities, 219 patients (31.3%) had systemic arterial hypertension and 41 patients (5.9%) had diabetes type 2 (T2DM).

In the total population, fifty-four patients (7.7%) had an anamnesis of infectious disease, fifty-eight patients (8.3%) of hematological disease, eight patients (1.1%) had a history of NSAID abuse, and fifteen patients (2.1%) of other nephrotoxic drugs.

Table 1 synthesizes the demographics and comorbidities of the general population in this study.

At the time of observation, 20.4% of patients (141/699) were diagnosed with one or more IRD. In particular, ninety-two patients had SLE, eleven patients APS, five patients RA, seven patients seronegative arthritis (SA), seven patients CV, ten patients other vasculitis, three patients SS, nine patients SSc, one patient myositis, one patient rheumatic polymyalgia, two patients primary immunodeficiency, and six patients other IRDs. The 1.7% of patients (12/699) had a diagnosis of an organ-specific IRD, such as inflammatory bowel disease (IBD) (five patients), thyroiditis (two patients), autoimmune cholangitis (AC) (three patients), T1DM (one patient), and celiac disease (one patient).

In the enrolled cohort, sixteen (2.3%) patients underwent a repeated kidney biopsy and two (0.3%) patients had a solitary functioning kidney. In addition, the most frequent complications of percutaneous kidney biopsy were gross hematoma in nine patients (1.3%) and arterio-venous fistula in ten patients (1.4%).

After performing kidney biopsies, a total of 176 patients (25.2%) had a kidney finding related to IRD.

**Figure 1:**
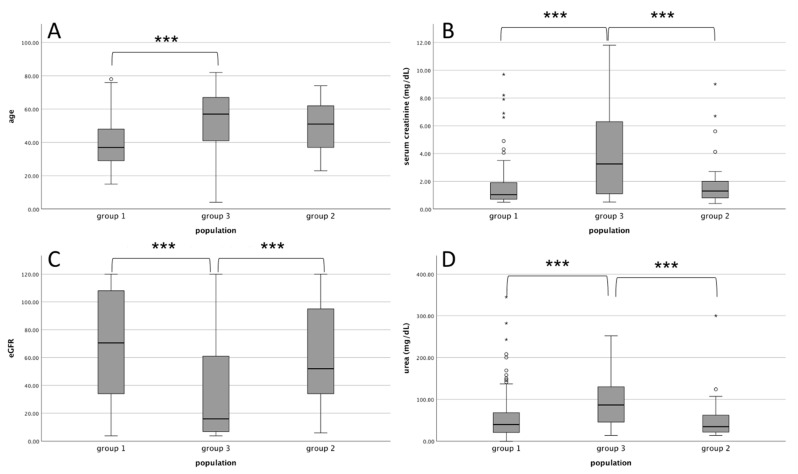
**Comparison of the study population split into three groups.** (**A**): age; (**B**): serum creatinine; (**C**): eGFR; (**D**): blood urea levels. Group 1: kidney histological correlated to IRD known at the time of biopsy. Group 2: kidney histological findings not correlated to IRD known at the time of biopsy. Group 3: “de novo” IRD not present in medical history at the time of biopsy. *** *p* < 0.001. IRD: immuno-rheumatological disease. eGFR: estimated glomerular filtration rate. circles and asterisks are outliers.

The study population was divided into three groups: patients with kidney histological findings correlated to underlying IRD known at the time of biopsy (Group 1), patients with kidney histological findings not correlated to underlying IRD known at the time of biopsy (Group 2), and patients with kidney histological findings compatible with “de novo” IRD that are not present in personal medical history known at the time of biopsy (Group 3). Figure 1 shows a graphical presentation of data between three groups according to age and kidney function.

In Group 1, there were 111 patients with kidney histological findings correlated to underlying IRD.

The diagnoses reported in patients’ anamneses were SLE in eighty-five patients, APS in ten patients, CV in seven patients, other vasculitis in three patients, SS in three patients, SSc in four patients, SA in three patients, RA in one patient, and other IRDs in five patients; eleven patients (9.9%) had more than one diagnosis of IRD.

No primary GN was found in Group 1. Secondary GN was diagnosed in 97% of the cases in Group 1 as follows: lupus nephritis (LN) in ninety-one patients; APS nephropathy (APSN) in six patients, two of them in association with LN; MPA in eight patients; and CV in five patients. TIN was diagnosed in two cases, specifically TIN related to IRD in one patient and one patient with IgG4 nephropathy. The VN diagnosis was present in three patients who had SRC. Table 2 synthesizes all features of Group 1.

In Group 2, there were 30 patients with kidney histological findings not correlated to underlying IRD. The diagnoses reported in the patients’ anamneses were APS (one patient), SA (four patients), RA (four patients), other IRD (one patient), vasculitis with no CV (seven patients), SLE (seven patients), myositis (one patient), SSc (four patients), and primary immunodeficiency (two patients). Some patients had more than one IRD.

In Group 2, according to kidney histological diagnoses, primary GN was found in twelve patients; membranous nephropathy (MN) was described in six patients, membranoproliferative glomerulonephritis (MPGN) in one patient, FSGS in two patients, IgAN in two patients, and MCD in one patient. Secondary GN was found in six patients, specifically, three patients with diabetic nephropathy, two patients with amyloidosis type A, and one patient who received a diagnosis of Anderson–Fabry disease. Vascular nephropathy was present in eight patients. One patient had minimal mesangial changes while one patient showed a kidney biopsy negative for pathological abnormalities. Table 3 synthesizes all features of Group 2.

In Group 3, there were 95 patients with kidney histological findings correlated to “de novo” IRD that were not present in their personal medical history.

**Figure 2:**
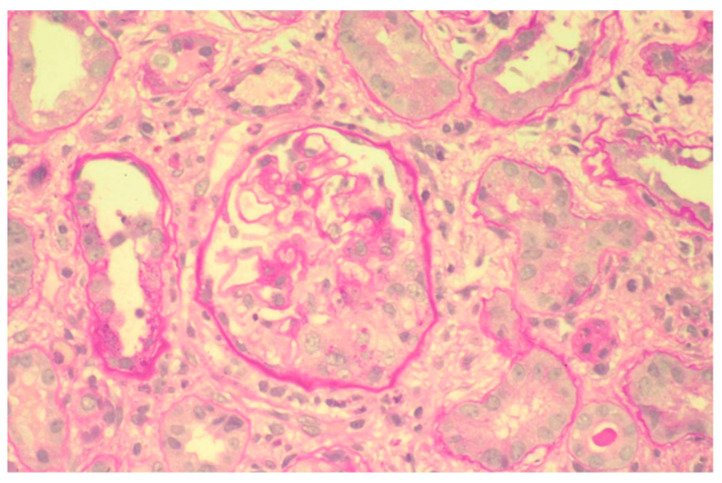
Proliferative extracapillary glomerulonephritis with the formation of a crescentic cell in the lower part of the glomerulus in microscopic polyangiitis (PAS). ×100.

At kidney biopsy, 98% of patients had a secondary GN. In particular, thirty-three patients had renal microscopic vasculitis, mainly represented by MPA (Figure 2), except for four patients with GPA and three patients with eosinophilic granulomatosis with polyangiitis (Churg–Strauss); twenty-four patients had LN; four patients CV; one patient PAN; and two patients APSN, one of them in association with LN. Tubular interstitial nephritis with a granulomatous pattern suggestive of sarcoidosis was found in one patient. Table 4 synthesizes all features of Group 3.

Regarding LN in all the patients enrolled, the classification into classes was as follows: nine patients were found in LN class II, twenty-eight patients in LN class III, sixty-eight patients in LN class IV, and ten in LN class V. In particular, in the “de novo” group, twenty patients were diagnosed with LN class IV, one patient with LN class III, and three with LN class V.

Then, to better understand clinical features and presentation of IRD and kidney involvement, features of Groups 1, 2, and 3 were compared. Table 5 and Table 6 show the comparative analysis between the groups.

Group 1 was characterized by a younger age than Group 3 (*p* < 0.001) and by a higher percentage of females than Group 2 and Group 3 (*p* < 0.001). Serum creatinine (*p* < 0.001) and urea (*p* < 0.001) values were significantly higher in Group 3 than in Group 1 and Group 2 while eGFR was lower (*p* < 0.001) in Group 3 compared to both Group 1 and Group 2. Thus, Group 3 showed a higher frequency of UA (*p* < 0.001), ANS (*p* < 0.001), and AKI (*p* < 0.001) than both Group 1 and Group 2. Finally, MPA was the most frequent diagnosis in Group 3 when compared with the other two groups (*p* < 0.001).

No other statistically significant differences were identified in the comparative analysis between the three groups.

## 6. Discussion

This is an observational study of patients who underwent native kidney biopsies performed across fifteen years focusing on IRD. Kidney involvement related to IRD was found in 25.2% of all biopsies performed. The kidney biopsy is the gold standard for diagnosing and starting specific therapy for glomerular diseases [14]. Several years ago, prior to the assessment of the imaging-guide percutaneous biopsy, the diagnostic algorithm for kidney diseases was based on clinical and laboratory data, without histological confirmation. Generally, the final and confirmed diagnosis differs from the main clinical hypothesis in up to one-third of cases [15].

To date, kidney biopsy is considered overall a safe procedure if contraindications, such as uncontrolled high blood pressure and coagulation disorders, are considered. In a recent multicenter prospective study of 5304 biopsies, in which we have participated, the native kidney biopsy was associated with 5.1% of major complication events [4]. In our study, the most frequent complications were gross hematoma (1.3% of cases) and artero-venous fistula (1.4% of cases).

From kidney biopsy, it is possible not only to confirm the highlighted diagnosis but also to discover the grading of severity and/or acute lesions susceptible to prompt therapy and prognosticating short-term and long-term outcomes [15]. In fact, it is well known that specific histopathological lesions, such as cellular crescents, fibrinoid necrosis, and neutrophil infiltration, can predict renal flares in LN [16]. To better diagnose patients with LN, classification criteria have been devised and revised over the years from the consensus of eighteen members of an international nephropathologist working group dividing LN into classes based on histopathological lesions. Also, activity and chronicity indices are applied to all classes together with new definitions of mesangial hypercellularity, cellular, fibrocellular, and fibrous crescents useful for driving immunosuppressive therapies [17].

Recently, in a multicenter Italian prospective study on percutaneous native kidney biopsies, among comorbidities in anamnesis, IRDs were found in 13.6% of patients while a confirmed diagnosis related to IRD was found in 14.3% of patients [4]. The difference found in the percentage of IRD in our study is mainly related to a specific referral center of IRD present in our hospital and with the aim of this study. In fact, Andrulli et al. [4] aimed to analyze the risks associated with percutaneous native kidney biopsies instead of epidemiologic studies describing the prevalence of detailed kidney diseases. However, in another Italian study about IRD kidney findings, crescentic GNs alone were reported in 21.6% [18]. Crescentic glomerulonephritis is characterized by crescents in 50% or more of the glomeruli for different causes resulting in rapidly progressive renal failure and most patients requiring renal replacement therapy. Based on the immunofluorescence microscopic pattern on the kidney biopsy, the crescentic glomerulonephritis is classified as a linear, granular, or pauci-immune histological finding. A linear pattern implies an anti-glomerular basement disease while granular staining is present in immune-complex-mediated diseases, such as lupus nephritis and post-infectious glomerulonephritis. Among kidney diseases that can share the same presentation are anti-glomerular basement-membrane glomerulonephritis, immunoglobulin A nephropathy, postinfectious glomerulonephritis, LN, Henoch–Schonlein purpura nephritis, and pauci-immune glomerulonephritis as MPA [19].

Our results showed some different features among groups of patients.

First, patients with the “de novo” IRD diagnosis at the kidney biopsy showed renal acute onset more than other groups. The main diagnoses reported in Group 3 were rapidly progressive glomerulonephritis and AKI presentation, which are mostly represented by vasculitis, mainly MPA.

It is well known that kidney injury is common in small vessel vasculitis, representing the most important predictor of mortality, and impacts the renal and global prognosis. In patients with MPA, the clinical presentation is mostly represented by AKI, caused by the formation of crescents observed in renal biopsy. Acute kidney injury is common in clinical practice and potentially treatable if detected early, improving prognosis and outcome. Acute kidney injury is defined in relation to creatine increase considering the time [11]. Recently, a new definition called “acute kidney disease” that includes functional and structural parameters as specific markers of kidney damage, like UA, has been validated [20]. The KDIGO Consensus Conference suggests that, when it is not possible to perform a kidney biopsy, immunologic tests can help to diagnose several parenchymal kidney diseases, such as glomerular/vascular diseases accounting for 10% of all acute kidney diseases requiring urgent care.

In patients with ANS onset and eGFR < 50 mL/min, the risk for death and lack of renal recovery is about 50% at 5 years [21].

Kidney involvement in vasculitis is characterized by extra capillary proliferation in the absence of the significant deposition of immunoglobulins associated with the rapid decline of GFR, hematuria, mild proteinuria, and hypertension over days or up to a few months [21]. Systemic small vessel vasculitis with renal involvement is mostly associated with an anti-neutrophil cytoplasmic antibody (ANCA) directed at leukocyte proteinase 3 (PR3-ANCA) and myeloperoxidase (MPO-ANCA), including MPA, GPA, and eosinophilic granulomatosis with polyangiitis (Churg–Strauss).

The primum movens in IRD-ANCA vasculitis is due to ANCA-activated neutrophils that adhere and penetrate microcirculation walls with the release of inflammatory factors that can activate the alternative complement pathway, increasing the inflammation pathway leading to fibrinoid necrosis in small vessels and crescents in glomeruli [22].

Although the epidemiology of ANCA-associated vasculitis is difficult due to the rarity of the autoimmunity disease, over time, a trend towards increasing incidence has been reported. A wide range of non-specific systemic symptoms, beyond insidious and non-symptomatic kidney injury, often delay the early recognition of vasculitis. Therefore, a delayed diagnosis remains a major concern for patients with vasculitis of which earlier management could improve renal outcome and overall survival [23]. Also, patients with “de novo” IRD at kidney biopsy showed a more advanced age and fewer females than patients with kidney histological findings correlated to underlying IRD. In ANCA-associated vasculitis, incidence increases with age, with a peak in the 60 to 70year age range with a slight male preponderance [21].

The more common clinical features, beyond anemia and fatigue, are related to the involvement of different organs, such as the skin (purpuric rash); lung (nodules, alveolar hemorrhage, asthma); nervous system (peripheral neuropathy, mononeuritis multiplex); cardiovascular system (myocarditis); ear, nose, and throat system (nasal crusting, epistaxis, sinusitis, nasal ulceration, septal perforation, subglottic stenosis, hearing loss); visual system (episcleritis, orbital granuloma, pseudo-tumor); and, obviously, kidney [24].

Although most cases of MPA can be linked to systemic complications and symptoms, some cases involve only the kidneys defining renal-limited vasculitis [25].

Despite the hallmark of small vessel vasculitis remains the ANCAs; in up to 30% of cases in pauci-immune renal vasculitis, ANCAs are absent [26]. However, in a large cohort of patients with small vessel vasculitis and renal involvement, the probability of ANCAs being negative, after the revision of the kidney biopsy by the Chapel Hill group, raised up to 30% when the intensities of staining for immunoglobulins were 0 and 1+, respectively (on a scale of 0 to 4+) [27]. Some authors believe that ANCA-negative renal vasculitis might be a distinct disease different from ANCA-positive vasculitis [28]. In fact, Chen et al. found that in eighty-five patients with MPA, twenty-eight (32.9%) of the eighty-five were ANCA negative both in immunofluorescence assay and ELISA. The authors observed higher proteinuria (5.47 ± 3.32 g/24 h versus 2.23 ± 2.27 g/24 h) and NS (46.4% versus 8.8%) in ANCA-negative patients. In addition, the prevalence of extrarenal manifestations was reduced in ANCA-negative patients vs ANCA-positive patients but the renal survival was significantly lower in ANCA-negative with respect to ANCA-positive patients [29]. On the other hand, some authors assume that it is important to remember that ANCA serum levels can fluctuate between negative and positive and, thus, all vasculitis can be ANCA positive [30]. Thus, the necessity to perform a kidney biopsy becomes mandatory to establish the diagnosis and target therapy.

In patients with kidney histological findings correlated to underlying IRD, most of the diagnoses are represented by LN. It is well known that new SLE cases occur in patients between 15 and 40 years of age, with incidence being dramatically higher in the female than in the male gender [23]. Also, LN is present in at least 50% of SLE cases, presenting a classic “full house” staining pattern with simultaneous positivity on immunofluorescence for IgA, IgG, IgM, C3, C1q, and light chains [31].

Usually, diagnosis of SLE precedes kidney involvement in most cases [32]. Rheumatologists must, therefore, be able to recognize new onset or recurrent LN and to refer to a specialist to perform a kidney biopsy. For this reason, the American College of Rheumatology recommends regular screening for new-onset and recurrent kidney involvement [33]. Since LN is categorized histologically into six classes by the International Society of okNephrology/Renal Pathology Society (ISN/RPS), as described above [17], with a wide range of clinical presentations from UA to ANS, patients with signs of new-onset LN should undergo a kidney biopsy to start a specific therapy early.

Also, the most frequent LN class in the “de novo” group was represented by class IV (83.3% of cases), characterized by active lesions, such as extracapillary or mesangiocapillary proliferation with capillary loop necrosis, karyorrhexis, and crescent. All these histological findings are supported by the acute onset of kidney injury, as shown in Group 3, with respect to the other groups.

Finally, the main difference from different groups was the lack of confirmation of hypothesized diagnoses before the biopsy and them not being confirmed after the biopsy, which changed the pharmacological treatment. In fact, in Group 2, unexpected secondary GN was found in six patients, including a diagnosis with diabetic nephropathy, amyloidosis type A, and Anderson–Fabry disease that requires different treatment and is not necessarily immunosuppressive.

The clinical hallmarks of kidney involvement used to diagnose GN are proteinuria, hematuria, and reduced GFR. The presence and/or severity of each of these signs depends on the underlying disease or risk factors [34].

Assessment of kidney function, also including urinalysis and ultrasound, especially in patients with a history of IRD, is periodically mandatory. Urinalysis, over the evaluation of eGFR, is a simple test that should be regularly assessed to identify red cells; granular, tubular, or mixed casts; active urinary sediment; or proteinuria that can provide a clue to detect early kidney involvement. Early referral to a nephrologist can confirm the organ involvement to limit the irreversible kidney damage with fibrosis and sclerosis development.

We acknowledge that our study has limitations, mainly related to the monocentric and retrospective study and the lack of specific autoantibodies required at the time of biopsy in all patients.

## 7. Conclusions

Cells of the immune system participate in the development of IRD with kidney involvement. Glomeruli, tubular, and vessels can be damaged, showing a different clinical presentation.

The onset of IRD with kidney involvement is often difficult to discover with a diagnostic delay. In addition, some IRDs could miss specific autoantibodies, such as ANCA, as in the case of renal-limited vasculitis.

Small-vessel ANCA-associated vasculitis and proliferative lupus are important causes of IRD that targets the kidney.

Although the clinical testing for autoantibodies is important, the relation between specific immunoglobulins and complement with IRD do not ensure a causal link.

Thus, the role of the kidney biopsy remains fundamental to discovering the onset of IRD, often with AKI, and to identifying another GN not closely related to the underlying IRD by starting targeted therapy.

## Figures and Tables

**Table 1:** General population in this study: demographics and comorbidities.

	Whole Cohort (*n* = 699)	Group 1 (*n* = 111)	Group 2 (*n* = 30)	Group 3 (*n* = 65)
Age, years, median (IQR)	48 (34–62)	37 (29–48)	51 (37–62)	57 (41–67)
Sex F, *n* (%)	364 (52.1)	92 (82.9)	15 (50)	42 (64.6)
Serum creatinine, mg/dL, median (IQR)	1.20 (0.80–2.30)	1.04 (0.70–1.90)	1.30 (0.80–2.00)	3.25 (1.10–6.30)
Serum urea, mg/dL, median (IQR)	41 (23–70)	40 (21–68)	35 (22–62)	86.50 (46–130)
eGFR, mL/min/1.73 m^2^, median (IQR)	54 (26–88)	70.5 (34–108)	52 (34–95)	16 (7–61)
Proteinuria, g/24 h, median (IQR)	2 (0.85–4.80)	1.61 (0.90–3.70)	1.00 (0.50–2.60)	1.20 (0.50–2.84)
UA, *n* (%)	280 (40.1)	57 (51.4)	17 (56.7)	16 (24.6)
ANS, *n* (%)	92 (13.2)	15 (13.5)	2 (6.7)	21 (32.3)
NS, *n* (%)	235 (33.6)	31 (27.9)	5 (16.7)	13 (20.0)
T2DM, *n* (%)	41 (5.9)	3 (2.7)	3 (10.0)	3 (4.6)
Systemic arterial hypertension, *n* (%)	219 (31.3)	16 (14.4)	10 (33.3)	17 (26.2)
Hematological diseases, *n* (%)	58 (8.3)	1 (0.9)	1 (3.3)	5 (7.7)
Infectious diseases, *n* (%)	54 (7.7)	4 (3.6)	3 (10.0)	7 (10.8)
NSAIDs abuse, *n* (%)	8 (1.1)	-	1 (3.3)	1 (1.5)
Other nephrotoxic drugs, *n* (%)	15 (2.2)	3 (2.7)	5 (16.7)	2 (3.1)

Group 1: patients with kidney histological findings correlated to underlying immuno-rheumatological diseases (IRDs); Group 2: patients with kidney histological findings not correlated to underlying IRDs; Group 3: patients with kidney histological findings compatible with “de novo” IRDs (absent in personal medical history). ANS: acute nephritis syndrome; eGFR: estimated glomerular filtration rate; F: female; IQR: interquartile range; NS: nephrotic syndrome; NSAIDs: non-steroidal anti-inflammatory drugs; T2DM: type 2 diabetes mellitus; UA: urinary abnormality.

**Table 2:** Anamnesis and kidney biopsy results of patients with kidney histological findings correlated to underlying immuno-rheumatological diseases (IRDs)—Group 1 (*n* = 111).

Anamnesis	*n* (%)
SLE	85 (76.6)
APS	10 (9.0)
CV	7 (6.3)
RA	1 (0.9)
SA	3 (2.7)
SSc	4 (3.6)
SS	3 (2.7)
other connective tissue diseases	5 (4.5)
non-CV vasculitis	3 (2.7)
polymyalgia	1 (0.9)
>1 autoimmune disease	11 (9.9)
myositis	-
primary immunodeficiencies	-
**Kidney biopsy diagnosis**	***n* (%)**
Primary GN	0
Secondary GN	108 (97.3)
APSN	6 (3.6) *
CV	5 (4.5)
LN	91 (81.9) *
MPA	8 (7.2)
TIN	2 (1.8)
TIN	1 (0.9)
IgG4	1 (0.9)
VN	3 (2.7)
SRC	3 (2.7)

* Two patients had both LN and APSN. APS: antiphospholipid syndrome; APSN: antiphospholipid syndrome nephropathy; CV: cryoglobulinemia vasculitis; GN: glomerulonephritis; LN: lupus nephropathy; MPA: microscopic polyangiitis; RA: rheumatoid arthritis; SA: seronegative arthritis; SLE: systemic lupus erythematosus; SRC: scleroderma renal crisis; SS: Sjogren syndrome; SSc: systemic sclerosis; TIN: tubulointerstitial nephritis; VN: vascular nephropathy.

**Table 3:** Anamnesis and kidney biopsy results of patients with kidney histological findings not correlated to underlying immuno-rheumatological diseases (IRDs)—Group 2 (*n* = 30).

Anamnesis	*n* (%)
SLE	7 (23.3)
APS	1 (3.3)
RA	4 (13.3)
SA	4 (13.3)
SSc	4 (13.3)
other IRD	1 (3.3)
non-CV vasculitis	7 (23.3)
>1 IRD	1 (3.3)
myositis	1 (3.3)
primary immunodeficiencies	2 (6.7)
**Kidney biopsy diagnosis**	***n* (%)**
Primary GN	12 (40)
FSGS	2 (6.7)
IgAN	2 (6.7)
MCD	1 (3.3)
MN	6 (20.0)
MPGN	1 (3.3)
Secondary GN	6 (20.0)
Anderson–Fabry disease	1 (3.3)
Diabetic glomerulosclerosis	3 (10.0)
RA-A	2 (6.7)
TIN	2 (6.7)
VN	8 (26.7)
NAS	8 (26.7)
No pathological changes and minimal mesangial abnormalities	2 (6.7)

APS: antiphospholipid syndrome; CV: cryoglobulinemia vasculitis; FSGS: focal segmental glomerulosclerosis; GN: glomerulonephritis; IgAN: IgA nephropathy; MCD: minimal change disease; MN: membranous nephropathy; MPGN: membranoproliferative glomerulonephritis; NAS: nephroangiosclerosis; RA: rheumatoid arthritis; RA-A: renal amyloidosis type A; SA: seronegative arthritis; SLE: systemic lupus erythematosus; SS: Sjogren syndrome; SSc: systemic sclerosis; TIN: tubulointerstitial nephritis; VN: vascular nephropathy.

**Table 4:** Anamnesis and kidney biopsy results of patients with kidney histological findings compatible with “de novo” immuno-rheumatological diseases (IRDs) (absent in personal medical history)—Group 3 (*n* = 65).

Kidney Biopsy Diagnosis	*n* (%)
Primary GN	0
Secondary GN	63 (98)
LN	24 (36.9) *
CV	4 (6.1)
** Renal microscopic vasculitis	33 (50.8)
PAN	1 (1.5)
APSN	2 (3.1) *
TIN	1 (1.5)
VN	0

* One patient had both LN and APSN. APSN: antiphospholipid syndrome nephropathy; CV: cryoglobulinemia vasculitis; GN: glomerulonephritis; LN: lupus nephritis; CV **. Renal microscopic vasculitis: twenty-six patients with microscopic polyangiitis (MPA); four patients with granulomatosis with polyangiitis; and three patients with eosinophilic granulomatosis with polyangiitis (Churg–Strauss). PAN: polyarteritis nodosa; TIN: tubulointerstitial nephritis; VN: vascular nephropathy.

**Table 5:** Comparative analysis between Group 1 and Group 3.

	Group 1 (*n* = 111)	Group 3 (*n* = 65)	*p* Value
Age, years, median (IQR)	37 (29–48)	57 (41–67)	<0.001
Sex F, *n* (%)	92 (82.9)	42 (64.6)	<0.001
Serum creatine, mg/dL, median (IQR)	1.04 (0.70–1.90)	3.25 (1.10–6.30)	<0.001
Serum urea, mg/dL, median (IQR)	40 (21–68)	86.50 (46–130)	<0.001
eGFR, mL/min/1.73 m^2^, median (IQR)	70.5 (34–108)	16 (7–61)	<0.001
Proteinuria, g/24 h, median (IQR)	1.61 (0.90–3.70)	1.20 (0.50–2.84)	>0.05
Hematuria, *n* (%)	75 (67.6)	39 (60)	<0.01
UA, *n* (%)	57 (51.4)	16 (24.6)	<0.001
ANS, *n* (%)	15 (13.5)	21 (32.3)	<0.001

Group 1: patients with kidney histological findings correlated to underlying immuno-rheumatological diseases (IRDs); Group 3: patients with kidney histological findings compatible with “de novo” IRD (absent in personal medical history). ANS: acute nephritis syndrome; eGFR: estimated glomerular filtration rate; F: female; IQR: interquartile range; UA: urinary abnormality.

**Table 6:** Comparative analysis between Group 2 and Group 3.

	[Group 2] (*n* = 30)	[Group 3] (*n* = 65)	*p* Value
Age, years, median (IQR)	51 (37–62)	57 (41–67)	>0.05
Serum creatine, mg/dL, median (IQR)	1.30 (0.80–2.00)	3.25 (1.10–6.30)	<0.001
Serum urea, mg/dL, median (IQR)	35 (22–62)	86.50 (46–130)	<0.001
eGFR, mL/min/1.73 m^2^, median (IQR)	52 (34–95)	16 (7–61)	<0.001
Proteinuria, g/24 h, median (IQR)	1.00 (0.50–2.60)	1.20 (0.50–2.84)	>0.05
Hematuria, *n* (%)	18 (60.0)	39 (60)	<0.01
UA, *n* (%)	17 (56.7)	16 (24.6)	<0.001
ANS, *n* (%)	2 (6.7)	21 (32.3)	<0.001

Group 2: patients with kidney histological findings not correlated with underlying immuno-rheumatological diseases (IRDs); Group 3: patients with kidney histological findings compatible with “de novo” IRD (absent in personal medical history). ANS: acute nephritis syndrome; eGFR: estimated glomerular filtration rate; F: female; IQR: interquartile range; UA: urinary abnormality.

## Data Availability

Data are contained within the article.
